# Americanitis

**Published:** 1883-06

**Authors:** 


### AMERICANITIS.

Sir Charles W. Dilke, in his "Greater Britain," thought he noticed a tendency in the Caucasian native American to acquire the red Indian type of physiognomy. Mr. W. Mattieu Williams echoes this opinion, and has cited several pieces of evidence to show that a change in the direction mentioned is going on, and that it is a process of dessication produced by the dryness of our climate. Mr. R. A. Proctor asserts that, during his three visits to America, he lost about thirty pounds in weight, which he recovered on returning home. Mr. Williams' own son, after residing for some time in this country, became thin, lank-jawed and sallow, "displaying all the characteristic symptoms of what I cannot refrain from calling *acute Americanitis*," but began to recover immediately after returning home.

On one occasion, at the house of the late George Combe, at Edinburgh, some family portraits were brought out, including those of members who had remained at home, and photographs of members who had emigrated to America a generation before, and with them a portrait of Black Hawk. "We placed the chief on one side, the Edinburgh portraits on the other, and those of the descendants of the American emigrants between, and all agreed that the deviations from the original family type were in a direction toward that of the red Indian. Mr. Combe maintains that this is generally the case, and I agree with him in regarding the typical "native American"—that is, the descendant of early English settlers—as displaying physically (I do not say intellectually and morally) a notable degree of reversion—or rather deviation—toward the aboriginal type displayed in the best examples of red Indians—that is, the old fighting chiefs.—*Popular Science Monthly.*

